# Health literacy in Europe: comparative results of the European health literacy survey (HLS-EU)

**DOI:** 10.1093/eurpub/ckv043

**Published:** 2015-04-05

**Authors:** Kristine Sørensen, Jürgen M. Pelikan, Florian Röthlin, Kristin Ganahl, Zofia Slonska, Gerardine Doyle, James Fullam, Barbara Kondilis, Demosthenes Agrafiotis, Ellen Uiters, Maria Falcon, Monika Mensing, Kancho Tchamov, Stephan van den Broucke, Helmut Brand

**Affiliations:** 1 Maastricht University, Department of International Health/CAPHRI, Maastricht, the Netherlands; 2 Ludwig Boltzmann Institute Health Promotion Research, Vienna, Austria; 3 The Cardinal Wyszyński Institute of Cardiology, Warsaw, Poland; 4 University College Dublin, Dublin, Ireland; 5 Hellenic American University, Manchester, NH & Hellenic American College, Athens, Greece; 6 National School of Public Health, Athens, Greece; 7 Centre for Nutrition, Prevention and health Services, National Institute for Public Health and the Environment, Bilthoven, Netherlands; 8 University of Murcia, Department of Legal Medicine, Murcia, Spain; 9 NRW Centre for Health, Bielefeld, Germany; 10 Medical University, Faculty of Public Health, Sofia, Bulgaria; 11 Université Catholique de Louvain, Louvain-la-Neuve, Belgium

## Abstract

**Background**: Health literacy concerns the capacities of people to meet the complex demands of health in modern society. In spite of the growing attention for the concept among European health policymakers, researchers and practitioners, information about the status of health literacy in Europe remains scarce. This article presents selected findings from the first European comparative survey on health literacy in populations. M**ethods**: The European health literacy survey (HLS-EU) was conducted in eight countries: Austria, Bulgaria, Germany, Greece, Ireland, the Netherlands, Poland and Spain (*n *= 1000 per country, *n *= 8000 total sample). Data collection was based on Eurobarometer standards and the implementation of the HLS-EU-Q (questionnaire) in computer-assisted or paper-assisted personal interviews. R**esults**: The HLS-EU-Q constructed four levels of health literacy: insufficient, problematic, sufficient and excellent. At least 1 in 10 (12%) respondents showed insufficient health literacy and almost 1 in 2 (47%) had limited (insufficient or problematic) health literacy. However, the distribution of levels differed substantially across countries (29–62%). Subgroups within the population, defined by financial deprivation, low social status, low education or old age, had higher proportions of people with limited health literacy, suggesting the presence of a social gradient which was also confirmed by raw bivariate correlations and a multivariate linear regression model. **Discussion**: Limited health literacy represents an important challenge for health policies and practices across Europe, but to a different degree for different countries. The social gradient in health literacy must be taken into account when developing public health strategies to improve health equity in Europe.

## Introduction

Health literacy has gained importance on the European health agenda. Closely linked to empowerment, it can be defined as ‘the ability of citizens to make sound decisions concerning health in daily life—at home, at work, in health care, at the market place and in the political arena’.^1^ The concept of ‘health literacy’ was originally used in the United States and Canada, however, it is now being used internationally, not only in health care, but also within the public health context.^2^ This is exemplified by the inclusion of health literacy in European policy documents such as in the European Commission White Paper entitled ‘Together for Health’,^3^ the Vilnius Declaration on Sustainable Health Systems for Inclusive Growth in Europe, agreed to by health ministers during the Lithuanian Presidency of the European Union,^4^ the Health 2020 strategy of the World Health Organization Regional Office for Europe^5^ and the WHO publication Health literacy: the solid facts.^6^

However, in spite of growing attention being paid to the concept among European health policymakers, information about the status of health literacy in Europe remains scarce. While several studies have demonstrated the prevalence of limited health literacy across the world,^7^ population data on health literacy levels for the European Union (EU) have thus far remained unavailable. To address this shortcoming, a consortium of nine organisations from eight EU member states (Austria, Bulgaria, Germany, Greece, Ireland, the Netherlands, Poland and Spain) launched the European Health Literacy Project (HLS-EU) to conduct the first comparative European health literacy survey.^8^ Notable aims of the project included developing a model instrument for measuring health literacy and generating first-time data on health literacy across diverse populations in the EU to make a comparative assessment and to provide an empirical basis for European, national and regional health policies.

A systematic literature review of existing health literacy definitions and models resulted in an integrated definition of the concept as ‘the knowledge, motivation and competences to access, understand, appraise and apply health information in order to make judgments and take decisions in everyday life concerning health care, disease prevention and health promotion to maintain or improve quality of life throughout the course of life’.^9^ In addition, a conceptual model was developed that captures the most comprehensive evidence-based dimensions of health literacy with its main antecedents and consequences.^9^ In the definition and the model, health promotion is understood in the broad sense defined by the World Health Organization in the Ottawa charter.^10^ This health literacy definition and model served as a basis for developing a multidimensional, comprehensive questionnaire to measure health literacy in the general populations; named the HLS-EU-Q.^11^

This article presents selected findings from the first European comparative survey using the HLS-EU-Q conducted in 2011. More in-depth descriptions of methods and results are available in the research report of the HLS-EU project.^12^ The paper will specifically consider how health literacy is distributed in the population of the countries involved, what proportions of the population show limited health literacy, which vulnerable groups have an above-average proportion of limited health literacy and whether there is a social gradient for health literacy.

## Methods

### Questionnaire development

Starting from the conceptual model of health literacy,^9^ a Delphi process among the HLS-EU Consortium members was conducted to generate items for assessing health literacy: the way people access, understand, appraise and apply information to make decisions regarding health care, disease prevention and health promotion. The resulting draft questionnaire was pre-tested for face validity in three focus groups (in Greece, Ireland and the Netherlands) and field-tested with 50 computer-assisted face-to-face interviews in two countries (*n *= 99 in Ireland and the Netherlands). Following the results of a principal component analysis and reliability analysis of the data, as well as inputs from consultations with external experts, a pre-final version of the questionnaire was achieved through a consensus-based item selection process. The pre-final version was subjected to a ‘plain language’ assessment by literacy experts to obtain the final version, which is known as HLS-EU-Q47 because it includes 47 items across 12 subdomains. For each item, respondents rated the perceived difficulty of a given task on a four-category Likert scale (i.e. very easy, easy, difficult and very difficult). This kind of operationalisation follows the tradition of subjective assessments of health literacy^13^ and reflects the interactive or relational nature of health literacy by measuring the fit of personal competences with contextual or situational demands of social systems.^14^ More details about the questionnaire’s development and the specific items of the HLS-EU-Q47 are presented in Sorensen et al.^11^

For the purpose of the HLS-EU survey the HLS-EU-Q47 was supplemented with an additional section, which contained 39 items referring to antecedents and precedents outlined in the conceptual model.^9^ They included inter alia indicators for the respondents’ health service use, health behaviour, subjective health status and socio-demographic and socio-economic situations supplemented with the Newest Vital Sign, which is a quick assessment of literacy.^15^ Examples of items include gender, age, education (using score cards and answers were transformed to international standard classification of education (ISCED) levels, social status, financial deprivation, self-assessed health (SF-36), long term illness, visit to general practitioner, hospital admission, alcohol consumption, smoking, exercise, work experience in health sector and insurance coverage. The final version of the questionnaire for the HLS-EU survey included the 47 health literacy items and the additional 39 items and was named the HLS-EU-Q86.

### Translation

The HLS-EU-Q86 was translated from English into six languages (Bulgarian, Dutch, German, Greek, Polish and Spanish) by professional translators and verified by the national research teams as well as by translators associated with TNS opinion, who facilitated the data collection on behalf of the HLS-EU Consortium.^12^

### Sampling

The HLS-EU survey was conducted as a population study according to Eurobarometer standards. A multistage random sampling procedure was applied in conformity with the sampling and inclusion criteria of the Eurobarometer methodology^12^^,^^16^ to draw an independent sample of 1000 persons aged 15 years and over from each of the 8 countries. Randomly selected sampling points were used from each administrative region in a country, stratified for regions with different population sizes and population densities (metropolitan, urban and rural areas).

Two exemptions were made for logistical and cost-efficiency reasons. Germany was only represented by its most populated federal state, North-Rhine Westphalia, which has a population of about 18 million people. In Greece, following general Eurobarometer practice, the survey collected data in greater Athens, a region with about 4 million people.

### Data collection and weighting

Data was collected in July and August 2011 by the international survey agency (TNS opinion), using either computer-assisted personal interviews (CAPI) or, in Bulgaria and Ireland, paper-assisted personal interviews (PAPI). Response rates differed significantly and were higher for countries where PAPI was used (75% in Bulgaria, 69% in Ireland) than in countries where CAPI was used (67% in Austria, 67% in Poland, 65% in Greece, 62% in Spain, 53% in Germany and 36% in the Netherlands). The considerably lower response rate for the Netherlands is probably associated with a difference in recruitment procedures: in accordance with local customs, Dutch participants were pre-recruited by phone or email to make appointments for interviews in people’s homes, rather than approached directly as in the other countries.^12^ To control for selection bias introduced by sampling and recruitment procedures, national datasets were weighted based on the most recent available national census data, using demographic Eurobarometer standard weights. Weighting criteria were age groups and gender (interlocked), regions [Nuts II regions (NUTS—Nomenclature of territorial units for statistics, as used by the statistical office of the European Union (EUROSTAT))] and size of municipality.

### Construction of the HLS-EU-Q47 health literacy indices

Using the scores on the 47 items measuring health literacy a comprehensive general index of health literacy was constructed. For that purpose, mean-based item raw scores were computed for respondents who gave valid answers to at least 80% of all health literacy questions (which was 96.2% of the total population of all sample countries tested). To simplify comparisons between scores on the general health literacy index and its various sub-indices, all scores were transformed to a unified metric with a minimum of 0 and a maximum of 50, where 0 represents the ‘least possible’ and 50 represents the ‘best possible’ health literacy score.

Following common practice for health literacy measures,^13^ index thresholds were defined and ranges for different levels of health literacy were created. Thresholds were set according to expert assessments of the required health literacy scores, which increase the likelihood of a person successfully pursuing his or her health interests. Threshold selection was performed in such a way that the correlation patterns between the resulting health literacy levels and important covariates deviated only minimally from those of the metric health literacy scores, while the correlation between level and metric score was maximised. The resulting four levels were ‘inadequate’ (0–25), ‘problematic’ (>25–33), ‘sufficient’ (>33–42) and ‘excellent’ (>42–50) health literacy. To detect vulnerable groups, the ‘inadequate’ and ‘problematic’ levels were combined to a single level, called ‘limited health literacy’ (0–33).

### Statistical analysis

Generally, results are presented for the eight participating countries in the comparative study and for the total sample. In order to have a valid country benchmark, the total sample was not weighted further by country size. Besides means and standard deviations for the index, percentage distributions were calculated for levels of limited health literacy for vulnerable groups. A multivariate linear regression model (sum of squares type III, missing values excluded list-wise) was used with the total sample to measure the effects of selected social determinants on health literacy.

## Results

### Distribution of health literacy

As shown in Table 1, the distribution of the health literacy indices for both the total sample and all national samples are unimodal and principally bell-shaped, yet with a consistently negative skew, particularly for Greece and Spain. In addition, the means are shifted towards the upper end of the scale. Both phenomena indicate a higher sensitivity of the measure for lower health literacy levels than for higher ones.

**Table 1:** Descriptive statistics of general health literacy index by country and for the total sample

Country	N	Min.	Max.	Mean	Std. Error	Std. Deviation	Skewness	Std. Error	Kurtosis	Std. Error
Austria	979	3.19	50	31.95	0.24	7.63	−0.07	0.08	−0.02	0.16
Bulgaria	925	0.00	50	30.50	0.30	9.17	−0.15	0.08	−0.01	0.16
Germany	1045	7.09	50	34.49	0.24	7.87	−0.01	0.08	−0.43	0.15
Greece	998	3.55	50	33.57	0.27	8.48	−0.54	0.08	0.57	0.16
Ireland	959	11.59	50	35.16	0.25	7.79	−0.17	0.08	−0.25	0.16
Netherlands	993	2.48	50	37.06	0.20	6.40	−0.12	0.08	−0.21	0.16
Poland	921	0.00	50	34.45	0.26	7.98	−0.39	0.08	0.95	0.16
Spain	974	15.60	50	32.88	0.20	6.10	0.42	0.08	0.51	0.16
Total	7795	0.00	50	33.78	0.09	7.95	−0.26	0.03	0.29	0.06

Mean health literacy scores varied considerably between countries, with a difference of 6.56 points (standardised mean difference = 0.80) between the countries with the highest (the Netherlands) and lowest (Bulgaria) mean health literacy scores. Compared to the total sample, higher mean values were observed for Ireland, Germany and Poland, but the mean value for the Netherlands was significantly (*P* < 0.01) higher than for any of the other surveyed countries. Standard deviations also varied remarkably, with a tendency to be larger for countries with lower health literacy averages (except for Spain). This indicates that some countries not only have lower health literacy on average, but also more inequality in terms of the distribution of health literacy in their population.

### Proportion of low health literacy in the population

**Figure 1:**
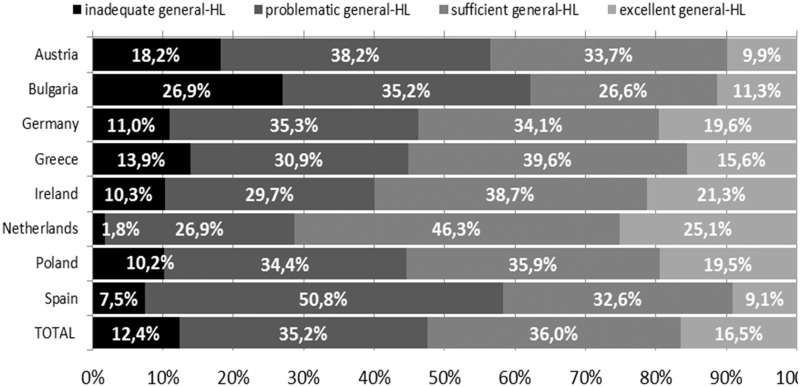
Levels of general health literacy index by country and for the total sample (HL: health literacy)

In the total sample, at least 1 out of 10 participants (12.4%) had inadequate health literacy. However, the differences between member states are substantial: only 1.8% of the sample in the Netherlands had inadequate health literacy, compared to 26.9% in Bulgaria (figure 1). Almost every second respondent (47.6%) in the total sample had limited (inadequate or problematic) health literacy, with the prevalence ranging from 28.7% in the Netherlands to more than 62.1% in Bulgaria.

### Groups who are vulnerable to having limited health literacy

As shown in Table 2, there are specific subgroups where the proportion of people with limited health literacy considerably exceeds the average (47.6%) observed for the overall sample. This holds true for people with poor health status, high use of health care services, low socio-economic status, lower education and older age. The highest proportion of limited health literacy was observed for people who reported a self-assessed health status of ‘very bad’ (78.1%) or ‘bad’ (71.8%), for those with more than one long-term illness (61%) and for those reporting six or more doctor visits in the last 12 months (58.9%). Therefore, worse health and thus higher demands for health services seems to be accompanied by lower levels of health literacy.

**Table 2:** Percentages of individuals with limited health literacy in selected vulnerable groups for countries and for the total sample

		Austria	Bulgaria	Germany	Greece	Ireland	Netherlands	Poland	Spain	Total
Health	Very bad	100.00	87.80	54.90	88.30	49.50	47.40	77.20	94.80	78.10
	Bad	84.20	82.40	54.90	80.30	57.20	41.40	71.20	75.30	71.80
Long term illness	Yes more than one	78.50	83.30	58.50	73.80	45.30	32.60	54.30	69.50	61.00
Doctor visits	6 times or more	70.00	74.00	56.20	58.40	46.60	30.80	54.10	69.40	58.90
Age	76 or older	72.60	75.40	53.90	72.30	46.00	28.80	65.50	71.10	60.80
	Between 66 and 75	71.40	79.70	39.70	66.20	37.10	30.40	58.70	77.10	58.20
Education	Levels 0 or 1	62.20	75.40	58.90	77.30	49.10	40.40	91.90	74.20	68.00
	Level 2	69.70	77.60	57.10	55.80	52.00	35.00	59.60	59.70	57.20
Problems with paying bills	Most of the time	67.10	75.20	46.80	60.70	61.20	33.50	42.20	61.70	63.40
Social status	Very Low	78.50	79.70	58.80	79.50	64.00	49.90	59.80	84.30	73.90
	Low	59.40	62.10	63.90	57.40	53.30	48.40	63.80	59.20	60.00

**Table 3:** Multivariate linear regression model for general health literacy index as dependent variable and socio-demographic indicators as predictors

	Coefficients	Standardised coefficients			95% confidence interval for B		Pearson correlations		
	B	Beta	t	Sig.	Lower bound	Upper bound	Raw correlation	Partial correlation	Semi-partial correlation
(Constant)	28.76		62.43	0.000	27.86	29.67			
Gender	1	0.06	5.86	0.000	0.67	1.33	0.05	0.07	0.06
Age	−0.04	−0.09	−8.52	0.000	−0.05	−0.03	−0.16	−0.10	−0.09
Education	0.79	0.13	11.33	0.000	0.65	0.93	0.25	0.13	0.12
Financial deprivation	−1.92	−0.24	−19.91	0.000	−2.11	−1.73	−0.34	−0.23	−0.21
Social status	0.69	0.14	11.39	0.000	0.57	0.81	0.31	0.13	0.12

With regard to socio-economic status, higher proportions of people with limited health literacy are found among those whose social status is ‘very low’ (73.9%) or ‘low’ (60%), followed by those with the lowest or low levels of education (68 and 57.2%), those who have permanent problems paying bills (63.4%), and those who are between 66 and 75 years old (58.2%) or 76 years or older (60.8%). Again, there are marked differences between countries. In some countries, the proportions of people with limited health literacy often exceeded 75% for certain vulnerable groups, whereas in the Netherlands the proportions generally stayed below 50%.

### Social gradient for health literacy

The finding that specific social groups contain higher proportions of people with limited health literacy, as described above, also suggests the existence of a social gradient for health literacy. This is confirmed by the substantial raw (bivariate) correlations between health literacy and selected possible social determinants. The raw correlation is strongest for financial deprivation (*r *= −.34), whereas the negative sign of the correlation indicates lower health literacy when financial deprivation increases. For social status, the raw correlation of health literacy (*r *= .31) is almost as strong, followed by education (*r *= .25), age (*r *= −.16; health literacy worsens with age) and gender (*r *= .05; men tend to have slightly lower health literacy). This kind of cross-sectional study comparing age-cohorts not only measures effects of aging, but also differences of generations.

However, as these social determinants are inter-correlated, performing a multivariate linear regression and controlling for possible covariates gives a better assessment of the direct effects these factors have on health literacy. A multivariate model—with all five social indicators introduced as independent variables—yielded an adjusted *R*^2^ = 17.4% (*P *= .000) for explained variance in health literacy. Financial deprivation remains the strongest predictor of low health literacy, followed by social status, education, age and gender.

## Discussion

The HLS-EU project is the first study to provide population data on health literacy at the EU level and to enable a comparison of health literacy levels between selected member states. It used a standard survey questionnaire based on a comprehensive conceptual and logic model, applied Eurobarometer standards^9^^,^^11^^,^^12^^,^^17^ and ensured consistency in data collection by using one European-wide represented agency.

Whereas the results indicate that more than 10% of the total surveyed population had an inadequate level of health literacy, this proportion varied between 1.8 and 26.9% by country. In turn, almost one in two citizens was affected when considering the proportion of limited health literacy (which varied between 29 and 62%). The considerable proportions of people with limited or inadequate health literacy imply that the health literacy deficit is a challenge for public health in European countries. Moreover, across countries, specific subgroups of the population have a higher proportion of people with limited health literacy than the general population, suggesting the existence of specific vulnerable groups, in addition to the presence of a social gradient in health literacy that is also confirmed by the survey results. Financial deprivation is the strongest predictor of low health literacy, followed by social status, education and age; whereas gender has a minor effect. As such, the HLS-EU data extends the well-documented phenomenon of a social gradient for health and for literacy.^16^ Given the richness of the HLS-EU data set, a much more detailed analysis is possible and is partly already being undertaken at both the national and comparative levels.^12^^,^^18^

However, it is also important to acknowledge the limitations of the study and its design. Due to limited financial resources, field testing for the HLS-EU survey was limited to three countries, the survey was carried out in only 8 of 27 EU member states, and the sample size was restricted to 1000 respondents for each sample country. Moreover, non-EU citizens living in the participating countries were left out of the survey in accordance with the Eurobarometer methodology. Differences between geographical representations within countries (Germany and Greece) and differences related to the data collection methodology (CAPI vs. PAPI; pre-recruiting) and response rates by country, partly limit strict comparability between countries. It is also important to note that the HLS-EU-Q47 is a subjective measurement and as such it does not include any objective items to measure functional health literacy. Noticeable the Newest Vital Sign was only included for comparison reasons in the HLS-EU-Q86 in the HLS-EU survey. Nevertheless, this first European comparative assessment provides important insights into how health literacy levels vary considerably both within and between the EU member states. To better understand the causes of the national differences, more analysis and specific further research is necessary. Apart from a few items, the measure seems generalisable within a European setting and its flexible matrix structure allows it to be adapted to suit national needs.

In conclusion, the HLS-EU survey has extended the evidence base on health literacy by measuring health literacy in eight EU member states. Limited health literacy and a social gradient in health literacy represent important challenges for health policies and practices in the EU, but to a different degree for participating member states. This health literacy deficit and inequality needs to be addressed by European and national health planners and policymakers who are dealing with the social determinants of health and health inequalities, and developing appropriate public health and health promotion strategies.

To that effect, a two-sided approach must be pursued, as recommended by Parker and Ratzan: (i) strengthen citizens’ and patients’ personal knowledge, motivation and competences to take well-informed health decisions; and (ii) decrease the complexity of society as a whole, and of the health care system in particular,^14^ so as to better guide, facilitate and empower citizens to sustainably manage their health.^4^^,^^5^^,^^16^ Efforts must be made to strengthen citizens’ health literacy by redesigning user-friendly and user-involving systems,^19^ adjusting curricula and training health professionals to better meet the challenge of the health literacy deficit, and increasing patients’ expectations of being active partners in their care. Due to the considerable differences in health literacy status between the countries, such measures need to be tailored towards a country’s specific social, economic, cultural and educational situation. At the EU level, this data provides possibilities for comparison, exchanging, benchmarking and learning from best practices.

For the latter, the HLS-EU-Q47 survey tool can be very useful for identifying strengths and weaknesses in health literacy levels, both within countries and in comparison with other countries. This questionnaire, which was based on a well thought out conceptual model of health literacy and validated on a large, cross-national sample of EU citizens following the well-established Eurobarometer methodology,^17^ allows for a reliable and valid measurement of health literacy and its components. By regularly monitoring health literacy, extending the number of countries that use the survey tool and integrating it in the EU’s health reporting and monitoring system, this data can significantly support political and professional decision-making to improve health literacy in Europe and, hence, contribute to the further improvement of the population’s health.
